# Magnitude of Stunting and Its Determinants Among Under‐Five Children in Awi Zone, Northwest Ethiopia

**DOI:** 10.1155/jnme/5984957

**Published:** 2026-02-10

**Authors:** Nigussie Adam Birhan, Kefale Tilahun Getahun, Zelalem Meraf Wolde, Tilahun Yimanu Birhan, Tess Astatkie

**Affiliations:** ^1^ Department of Statistics, College of Natural and Computational Science, Injibara University, Injibara, Ethiopia, inu.edu.et; ^2^ Department of Epidemiology and Biostatistics, Institute of Public Health, College of Medicine and Health Science, University of Gondar, Gondar, Ethiopia, uog.edu.et; ^3^ Faculty of Agriculture, Dalhousie University, Truro, Nova Scotia, Canada, dal.ca

**Keywords:** Awi Zone, determinants, prevalence, stunting, under-five children

## Abstract

**Introduction:**

Stunting is a major global health concern, with an estimated 154.8 million children under 5 years old. This study aimed to assess the prevalence and the contributing factors of stunting among children under 5 years old in Awi Zone, Northwest Ethiopia.

**Methods:**

A community‐based cross‐sectional study was conducted on 1388 participants selected using a multistage sampling method. Structured and pretested questionnaires were used to collect the data, and STATA software was used for analysis. Multivariable logistic regression analysis was performed to identify the factors associated with stunting.

**Results:**

The prevalence of stunting in Awi Zone was 24.9%. Rich wealth index (AOR: 0.46), health insurance (AOR: 0.45), child aged 24–59 months (AOR: 30.40), medium birth weight (AOR: 0.53), having toilet facility (AOR: 0.46), male child (AOR: 1.92), 2^nd^ or 3^rd^ birth order (AOR: 1.99), institutional delivery (AOR: 0.48), exclusive breastfeeding (AOR: 0.11), and ANC (AOR: 0.64) are significantly associated with stunting.

**Conclusion:**

To reduce child stunting, governmental and nongovernmental organizations should design and implement public health interventions targeting poor households and those without health insurance and should enhance institutional delivery and ANC visits.

## 1. Introduction

Childhood malnutrition is determined by the complex interactions between food consumption, poor sanitation, overall health, and healthcare practices [1, 2]. Undernutrition can be caused by inadequate food intake, poor nutrient utilization due to illnesses, or a combination of these factors [3]. Stunting, defined as a low height‐for‐age Z‐score (HAZ), is a widespread indicator of chronic undernutrition that impairs children’s linear growth and remains a major global public health challenge [4, 5].

Numerous factors, including environmental factors, infectious diseases, micronutrient deficiencies, maternal nutrition, and socioeconomic level, can induce stunting in children, which can occur within the first 1000 days of conception [6, 7]. Stunting has serious long‐term effects on physical and cognitive development, serving as a key indicator of a country’s nutritional and health status, and is linked to poor educational outcomes, lower productivity in maturity, and a higher risk of chronic diseases later in life [8, 9].

Globally, an estimated 154.8 million children under 5 years old are stunted [10]. In Ethiopia, the costs associated with undernourished children have been estimated at 55.5 billion Ethiopian birr (1.51 billion USD), which is equivalent to 16.5% of the gross domestic product (GDP) [11]. According to the 2016 Ethiopia Demographic and Health Survey (EDHS), 38% of children under five are stunted, which is significantly higher than the global average of 22.9% [12].

Previous national and regional studies have identified individual‐ and household‐level determinants of stunting in Ethiopia, such as poor maternal health and nutrition, low maternal education, inadequate infant and young child feeding practices, low birth weight, short birth intervals, small size of the child at birth, and a lack of access to healthcare [13, 14]. However, previous studies have focused on broad national or regional patterns, and there is limited evidence on the local‐level prevalence and determinants of stunting in Awi Zone, which is characterized by diverse agro‐ecological conditions, socioeconomic disparities, and varying access to health and nutrition services. Understanding such local‐level differences is crucial for designing targeted and efficient nutrition interventions. Therefore, this study aimed to analyze the determinants of stunting among children under five in Awi Zone, Northwest Ethiopia, to provide area‐specific evidence for policy and programmatic actions.

## 2. Materials and Methods

### 2.1. Study Design, Setting, and Period

The community‐based cross‐sectional study design was conducted from February to June 2023 in Awi Zone, Amhara Region, Ethiopia. The zone has an estimated population of 982,942 of whom 491,865 are men, and 491,077 are women, divided into six urban and nine rural districts [15]. It is located 114 km from the capital city of the Amhara Region (Bahir Dar) and 449 km from the capital city of Ethiopia (Addis Ababa). It has 125 private clinics, 46 health centers, and 5 public hospitals; only one private hospital provides preventive and curative services to the community [16].

### 2.2. Study Population

The population comprised under‐five‐year‐old children in Awi Zone who were paired with their mothers/caregivers. Mothers/caregivers who resided in the research area for less than 6 months before data collection and severely ill children were excluded from this study.

### 2.3. Sample Size and Sampling Procedure

The sample size was determined using a single population proportion formula using 95% confidence level, 5% margin of error, and 0.27 prevalence of stunting in Ethiopia [17]. The formula resulted in 841. After considering a 10% nonresponse rate and a design effect of 1.5, the final sample size was *n* = 1388.

A multistage sampling method was employed, starting with a random selection of 8 of the 15 districts, then a random selection of 34 kebeles from the 8 districts, and then selecting 1388 households from the 34 kebeles using systematic random sampling.

### 2.4. Data Collection Tools and Procedures

A structured and pretested questionnaire was developed based on the EDHS and related literature. Mothers/caregivers were interviewed face‐to‐face, and children’s anthropometric measurements were taken. In households with multiple children under 59 months, the youngest was selected. Eight data collectors and two supervisors received 3 days of training about the study objectives, sample selection, interviewing, and ethical considerations. The original questionnaire was prepared in English and then translated into Amharic and then translated back into English to ensure consistency. A pretest was conducted on 5% of the sample in a nonstudy kebele to check for clarity, sequence, and cultural appropriateness. Necessary modifications were made accordingly. Children under 24 months were measured lying down, and those 24–59 months were measured standing. Measurements were taken twice to the nearest 0.1 cm, and the average was used.

### 2.5. Variables of the Study

The dependent variable is stunting, defined as HAZ below 2SD according to WHO standards, coded as 1 for stunted and 0 for not stunted [18]. The independent variables are as follows: place of residence (urban and rural), child’s sex (male and female), religion (Orthodox and other), mother’s education (no education, primary, and secondary+), marital status (married and other), mother’s occupation (employed and not employed), mother’s age (< 25, 25–34, and ≥ 35 years), birth order (first, 2nd–3rd, 4th–5th, and ≥ 6th), family size (≤ 5 and > 5), number of under‐five children (1 and ≥ 2), child’s age (< 6, 6–11, 12–23, and 24–59 months), wealth index (poor, middle, and rich), media exposure (yes and no), child’s birth weight (small, medium, and large), source of drinking water (protected and unprotected), toilet facility (yes and no), place of delivery (home and institutional), complementary feeding at six months (yes and no), exclusive breastfeeding (yes and no), health insurance (yes and no), antenatal care visit (yes and no), and diarrhea in the last two weeks (yes and no). The household wealth index was constructed using principal component analysis (PCA) based on household assets (radio, television, and livestock), housing materials, water source, and sanitation facilities.

### 2.6. Data Management and Analysis

The data were analyzed using STATA 18. The frequency and percentages of all variables were presented using descriptive statistics. A binary logistic regression model was used to describe the relationship between the dependent and the independent variables. Bivariate logistic regression analysis was done, and crude odds ratio values were used to identify the factors associated with stunting at a 5% significance level. In the multivariable logistic regression analysis, the adjusted odds ratio (AOR) values were used to determine the factors associated with stunting among children under five at a 5% significance level.

### 2.7. Ethics Approval and Consent to Participate

Ethical clearance was obtained from College of Natural and Computational Science Research and Community Service, Injibara University (Ref: CNCS/PG/R/CS/V/Dean/255/23). Permission was secured from district and kebele authorities. Written informed consent was obtained from all participants after explaining the study’s purpose. Confidentiality was maintained using unique household ID. Participation was voluntary, and the right to withdraw was respected. All methods were carried out in accordance with relevant guidelines and regulations.

## 3. Results

### 3.1. Prevalence of Stunting Among Under‐Five Children

**FIGURE 1:**
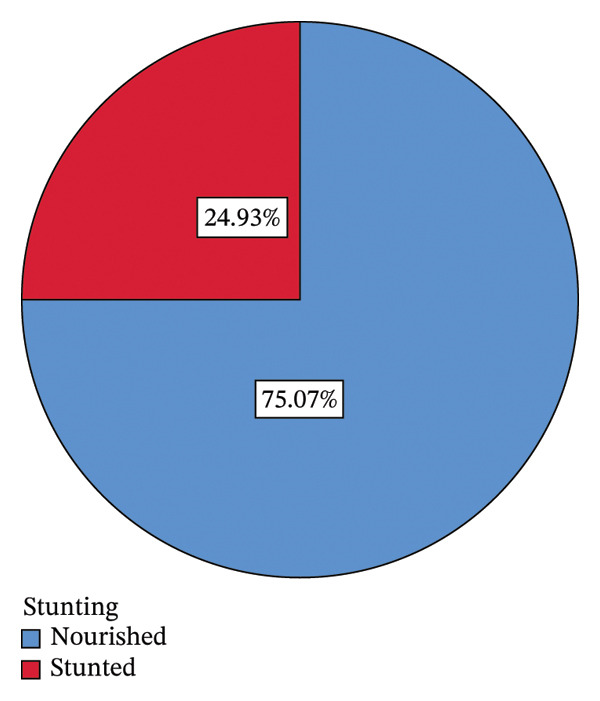
Prevalence of stunting among children under five in Awi Zone.

The findings of this study revealed that the prevalence of stunting among children under 5 years old in Awi Zone is 24.93% (Figure 1).

### 3.2. Sociodemographic Characteristics of the Study Participants

Majority (95.1%) of the mothers were married, and 53.67% were unable to read and write. Slightly more than half (56.41%) of the children were males, and 53.96% of the mothers/caregivers were in the age group of 25–34 years. 92.4% of them had toilet facilities, 24.2% of them had stunted children, and 16.1% of them used unprotected drinking water sources. 93.2% of them delivered their child at a health facility, and 61.1% of them gave supplementary food to their child at 6 months (Table 1).

**TABLE 1:** Descriptive statistics of child stunting by study characteristics.

Characteristics	Category	Frequency (%)	Stunting within category	
			Nourished (%)	Stunted (%)
Residence	Urban	474 (34.2)	374 (78.9)	100 (21.1)
	Rural	914 (65.9)	668 (73.1)	246 (26.9)

Gender	Male	783 (56.4)	611 (78.0)	172 (22.0)
	Female	605 (43.6)	431 (71.2)	174 (28.8)

Religion	Orthodox	1307 (94.2)	994 (76.1)	313 (24.0)
	Other^a^	81 (5.8)	48 (5.8)	33 (40.7)

Mother’s education	No education	745 (53.7)	544 (73.0)	201 (27.0)
	Primary school	351 (25.9)	272 (77.5)	79 (22.5)
	Secondary+	292 (21.0)	226 (77.4)	66 (22.6)

Marital status	Married	1320 (95.1)	998 (75.6)	322 (24.4)
	Other^b^	68 (4.9)	44 (64.7)	24 (35.3)

Mother’s occupation	No	76 (5.2)	54 (71.1)	22 (29.0)
	Yes	1312 (94.5)	998 (75.3)	324 (24.7)

Mother’s age	Under 24	125 (9.0)	104 (83.2)	21 (16.8)
	25–34	749 (56.0)	590 (78.8)	159 (21.2)
	35 and above	514 (37.0)	348 (67.7)	166 (32.3)

Birth order	First	338 (24.4)	265 (78.4)	73 (21.6)
	2^nd^ or 3^rd^	514 (37.0)	383 (74.5)	131 (25.5)
	4^th^ or 5^th^	327 (23.6)	243 (74.3)	84 (25.7)
	6^th^ or above	209 (15.1)	151 (72.3)	58 (27.8)

Family size	5 or less	626 (45.1)	481 (76.8)	145 (23.2)
	Above 5	762 (54.9)	561 (73.6)	201 (26.4)

Number of < 5 children	1	1072 (77.2)	824 (76.9)	248 (23.1)
	≥ 2	316 (22.8)	218 (69.0)	98 (31.0)

Child age	Less than 6	124 (8.9)	122 (98.4)	2 (1.6)
	6–11	208 (15.0)	204 (98.1)	4 (1.9)
	12–23	388 (28.0)	373 (96.1)	15 (3.9)
	24–59	668 (48.1)	343 (51.4)	325 (48.7)

Wealth index	Poor	570 (41.17)	396 (69.5)	174 (30.5)
	Middle	289 (20.8)	232 (80.3)	57 (19.7)
	Rich	529 (38.1)	414 (78.3)	115 (21.7)

Media exposure	No	721 (52.0)	517 (71.7)	204 (28.3)
	Yes	667 (48.1)	525 (78.7)	142 (21.3)

Childbirth weight	Small	160 (11.5)	83 (51.9)	77 (48.1)
	Medium	935 (67.4)	734 (78.5)	201 (21.5)
	Large	293 (21.1)	225 (76.8)	68 (23.2)

Source of drinking water	Unprotected	224 (16.1)	163 (72.8)	61 (27.2)
	Protected	1164 (83.9)	879 (75.5)	285 (24.5)

Toilet facility	No	105 (7.6)	69 (65.7)	36 (34.3)
	Yes	1283 (92.4)	973 (75.8)	310 (24.2)

Place of delivery	Home	95 (6.8)	62 (65.3)	33 (34.7)
	Institutional facility	1293 (93.2)	980 (75.8)	313 (24.2)

Complementary feeding at 6 months	No	540 (38.9)	378 (70.0)	162 (30.0)
	Yes	848 (61.1)	664 (78.3)	184 (21.7)

Exclusive breastfeeding status	No	462 (33.3)	180 (39.0)	282 (61.0)
	Yes	926 (66.7)	862 (93.1)	64 (6.9)

Health insurance	No	314 (22.6)	225 (71.7)	89 (28.3)
	Yes	1074 (77.4)	817 (76.1)	257 (23.9)

ANC	No	590 (42.5)	413 (70.0)	177 (30.0)
	Yes	798 (57.5)	629 (78.8)	169 (21.2)

Diarrhea	No	1155 (83.2)	880 (76.2)	275 (23.8)
	Yes	233 (16.8)	162 (69.5)	71 (30.5)

^a^Muslim and Protestant.

^b^Single, divorced, and widowed.

### 3.3. Associated Factors of Stunting

The results show that child’s age, child’s gender, birth order, number of under‐five children in household, wealth index of household, toilet facility, place of delivery, health insurance, child’s birth weight, exclusive breastfeeding status, and ANC have a statistically significant association with stunting (Table 2).

**TABLE 2:** Bivariable and multivariable logistic regression analysis of stunting and associated risk factors.

Characteristics	Category	COR	*p* value	AOR (95% CI)	*p* value
Child age	Less than 6	1		1	
	6–11	1.196	0.838	1.41 (0.24, 8.26)	0.706
	12–23	2.453	0.238	2.64 (0.55, 12.59)	0.223
	24–59	57.799	< 0.001	30.40 (6.86, 134.64)	< 0.001

Residence	Urban	1		1	
	Rural	1.377	0.018	0.98 (0.57, 1.66)	0.925

Gender	Female	1		1	
	Male	1.434	0.004	1.92 (1.34, 2.74)	< 0.001

Birth order	1^st^	1		1	
	2^nd^ or 3^rd^	1.242	0.194	1.99 (1.18, 3.37)	0.010
	4^th^ or 5^th^	1.255	0.215	0.59 (0.28, 1.23)	0.157
	6^th^ or more	1.394	0.102	0.71 (0.30, 1.67)	0.432

Religion	Orthodox	1		1	
	Other	2.183	0.001	1.99 (0.86, 4.58)	0.107

Number of < 5 children	1	1		1	
	≥ 2	1.494	0.005	3.57 (2.24, 5.69)	< 0.001

Mother’s education	No education	1		1	
	Primary school	0.786	0.114	1.00 (0.60, 1.65)	0.992
	Secondary+	0.790	0.148	0.95 (0.53, 1.71)	0.866

Marital status	Married	1		1	
	Other^∗a^	1.691	0.045	0.80 (0.35, 1.79)	0.579

Mother’s occupation	No	1			
	Yes	0.805	0.406	0.49 (0.38, 1.33)	0.282

Mother’s age	Less than 24	1			
	24–34	1.335	0.258	0.86 (0.41, 1.82)	0.694
	Above 34	2.362	0.001	1.80 (0.75, 4.36)	0.189

Family size	5 and less	1		1	
	Above 5	1.189	0.169	0.97 (0.60, 1.58)	0.916

Wealth index	Poor	1		1	
	Medium	0.559	0.001	0.92 (0.57, 1.50)	0.736
	Rich	0.632	0.001	0.46 (0.26, 0.83)	0.010

Media exposure	No	1		1	
	Yes	0.685	0.003	0.68 (0.45, 1.03)	0.068

Toilet facility	No	1		1	
	Yes	0.611	0.022	0.46 (0.24, 0.89)	0.020

Source of drinking water	Unprotected	1			
	Protected	0.866	0.384	0.77 (0.44, 1.39)	0.287

Place of delivery	Home	1			
	Institutional facility	0.600	0.023	0.48 (0.25, 0.91)	0.026

Complementary feeding at 6 months	No	1		1	
	Yes	0.647	0.001	0.91 (0.69, 1.44)	0.987

Health insurance	No	1		1	
	Yes	0.795	0.112	0.45 (0.27, 0.74)	0.002

Diarrhea	No	1		1	
	Yes	1.402	0.033	1.32 (0.84, 2.07)	0.229

Childbirth weight	Small	1		1	
	Medium	0.295	< 0.001	0.532 (0.319, 0.887)	0.016
	Large	0.326	< 0.001	0.795 (0.432, 1.464)	0.462

Exclusive breastfeeding status	No	1		1	
	Yes	0.047	< 0.001	0.109 (0.072, 0.164)	< 0.001

ANC	No	1		1	
	Yes	0.627	< 0.001	0.642 (0.441, 0.936)	0.021

Children aged 24–59 months had markedly higher odds of stunting compared to those under 6 months (AOR = 30.4, 95% CI: 6.86–134.6), reflecting the large difference in stunting prevalence across age groups. Male children have a 91.8% higher chance of being stunted compared to female children. Children of 2^nd^ or 3^rd^ birth order have twice the risk of stunting compared to firstborns. Children in households with two or more under‐five children have 3.57 times higher risk of stunting compared to those with only one under‐five child. Children from wealthier households and households with toilet facilities, health insurance coverage, institutional delivery, medium birth weight, exclusive breastfeeding, and ANC follow‐up had lower odds of stunting (Table 2).

## 4. Discussion

The prevalence of stunting in Awi Zone is 24.9%, which is comparable to findings in Uganda (24.2%) [19] and higher than that in Kenya (21.9%) [19] and Ghana (18.4%) [20], but lower than that in Niger (43.9%) [21] and Ethiopia (41%) [22]. These disparities may be due to geographic heterogeneity in risk factors, socioeconomic status, health infrastructure, and child nutrition.

Children aged 24–59 months have a higher chance of stunting than children aged less than 6 months. This is consistent with the studies conducted in Congo [23] and in Rwanda [24]. A plausible reason might be that children at this developmental stage can crawl and grasp any objects, increasing exposure to infections like diarrhea and pneumonia, which may lead to stunting [19, 25].

Children born in health facilities have lower odds of stunting, consistent with studies from Iran [26] and Ethiopia [27, 28]. This may be because institutional deliveries reduce stunting risk by providing immediate newborn care, facilitating early breastfeeding initiation, and promptly addressing complications.

Our study showed that children from rich families have lower odds of stunting, aligning with studies from India [29], Ethiopia [30], Indonesia [31], and Nigeria [32]. This may be due to wealthier families’ ability to purchase nutrient‐rich foods, access necessary health services during pregnancy, and provide better care due to higher educational levels [27]. Children with larger birth sizes were less likely to be stunted, which is similar to the findings from Nepal [33], Nigeria [34], and Tanzania [34], which may be driven by a lack of nutritional supplements during pregnancy and later after birth. Additionally, male children are at a higher risk of being stunted than female children, which is in line with findings in Nigeria [34], Tanzania [35], and China [36]. This early introduction of supplementary food and fluid predisposes male children to diarrhea and other infections; these obviously increase nutritional demand over reduced appetite. However, some studies have reported no significant sex differences in stunting [37, 38], suggesting that the association may vary by context, cultural practices, or measurement methods.

## 5. Strengths and Limitations of the Study

The strengths of this study include the use of multiple data collection methods, such as on‐site observation and measurement, along with a pretest to ensure data quality and consistency. However, a limitation of a cross‐sectional survey is that it cannot establish causality. Self‐reported data may also be subject to recall and social desirability bias, potentially affecting the accuracy of the responses. Furthermore, despite controlling for several known confounders, the possibility of residual or unmeasured confounding cannot be entirely excluded.

## 6. Conclusions

Age of the child, gender of the child, childbirth order, the number of children under five in the household, place of delivery, birth weight of the child, wealth index of the household, exclusive breastfeeding, antenatal care visit, toilet facility, and health insurance are significantly associated with stunting among children under 5 years in Awi Zone, Northwest Ethiopia. We recommend that mothers have exclusive breastfeeding practices, childcare, and infection prevention protocols. Therefore, to improve child nutrition status, governmental and nongovernmental organizations should design and implement public health interventions targeting those without health insurance, and enhancing institutional delivery is vital for reducing child stunting.

## Author Contributions

Nigussie Adam Birhan contributed to the design and supervision of data collection, performed data analysis and interpreted the results, and drafted the manuscript. Zelalem Meraf Wolde supervised data collection and evaluated the scientific content of the study. Kefale Tilahun Getahun and Tilahun Yimanu Birhan participated in designing the study, data analysis, and data interpretation. Tess Astatkie was involved in advising throughout each stage and reviewed and edited the manuscript. All authors agreed on the journal to which the article will be submitted and agreed to be accountable for all aspects of the work.

## Funding

The financial support was provided by Injibara University.

## Disclosure

The funding organization had no role in data collection, analysis, or write‐up of the report. All authors gave final approval of the version to be published.

## Consent

The authors have nothing to report.

## Conflicts of Interest

The authors declare no conflicts of interest.

## Data Availability

The data that support the findings of this study are available from the corresponding author upon a reasonable request.
